# Molecular High-Grade B-Cell Lymphoma: Defining a Poor-Risk Group That Requires Different Approaches to Therapy

**DOI:** 10.1200/JCO.18.01314

**Published:** 2018-12-03

**Authors:** Chulin Sha, Sharon Barrans, Francesco Cucco, Michael A. Bentley, Matthew A. Care, Thomas Cummin, Hannah Kennedy, Joe S. Thompson, Rahman Uddin, Lisa Worrillow, Rebecca Chalkley, Moniek van Hoppe, Sophia Ahmed, Tom Maishman, Josh Caddy, Anna Schuh, Christoph Mamot, Catherine Burton, Reuben Tooze, Andrew Davies, Ming-Qing Du, Peter W.M. Johnson, David R. Westhead

**Affiliations:** ^1^ University of Leeds, Leeds, United Kingdom; ^2^ St James’ University Hospital, Leeds, United Kingdom; ^3^ University of Cambridge, Cambridge, United Kingdom; ^4^ Cancer Research UK Centre and Southampton Clinical Trials Unit, University of Southampton, Southampton, United Kingdom; ^5^ University of Oxford, Oxford, United Kingdom; ^6^ Cantonal Hospital Aarau, Aarau/Swiss Group for Clinical Cancer Research, Switzerland

## Abstract

**Purpose:**

Biologic heterogeneity is a feature of diffuse large B-cell lymphoma (DLBCL), and the existence of a subgroup with poor prognosis and phenotypic proximity to Burkitt lymphoma is well known. Conventional cytogenetics identifies some patients with rearrangements of *MYC* and *BCL2* and/or *BCL6* (double-hit lymphomas) who are increasingly treated with more intensive chemotherapy, but a more biologically coherent and clinically useful definition of this group is required.

**Patients and Methods:**

We defined a molecular high-grade (MHG) group by applying a gene expression–based classifier to 928 patients with DLBCL from a clinical trial that investigated the addition of bortezomib to standard rituximab plus cyclophosphamide, doxorubicin, vincristine, and prednisone (R-CHOP) therapy. The prognostic significance of MHG was compared with existing biomarkers. We performed targeted sequencing of 70 genes in 400 patients and explored molecular pathology using gene expression signature databases. Findings were validated in an independent data set.

**Results:**

The MHG group comprised 83 patients (9%), with 75 in the cell-of-origin germinal center B-cell-like group. *MYC* rearranged and double-hit groups were strongly over-represented in MHG but comprised only one half of the total. Gene expression analysis revealed a proliferative phenotype with a relationship to centroblasts. Progression-free survival rate at 36 months after R-CHOP in the MHG group was 37% (95% CI, 24% to 55%) compared with 72% (95% CI, 68% to 77%) for others, and an analysis of treatment effects suggested a possible positive effect of bortezomib. Double-hit lymphomas lacking the MHG signature showed no evidence of worse outcome than other germinal center B-cell-like cases.

**Conclusion:**

MHG defines a biologically coherent high-grade B-cell lymphoma group with distinct molecular features and clinical outcomes that effectively doubles the size of the poor-prognosis, double-hit group. Patients with MHG may benefit from intensified chemotherapy or novel targeted therapies.

## INTRODUCTION

Aggressive B-cell non-Hodgkin lymphomas, including diffuse large B-cell lymphoma (DLBCL) and Burkitt lymphoma (BL), comprise a heterogeneous class of related malignancies for which response and survival on standard treatment vary substantially, with significantly worse outcomes in some subtypes. DLBCL incidence is high and carries a significant disease burden,^1^ whereas BL is a distinct and highly proliferative entity that requires substantially more intensive chemotherapy. Within DLBCL, the cell-of-origin (COO) variants germinal center B-cell-like (GCB) and activated B-cell-like (ABC) DLBCL have been defined by gene expression patterns.^2^ These have different underlying molecular pathology and prognosis, but internal heterogeneity in their genetic and phenotypic features indicates that further stratification is necessary for precision treatment.^3^

Several groups recently have considered DLBCL stratification by using integrated genetic information, providing prognostic models,^4^ or separating patients further into smaller subgroups on the basis of shared genetic features.^5,6^ Earlier work identified patients with key chromosomal rearrangements of *MYC* and *BCL2* and/or *BCL6* genes (double and triple hits) that correlated with poor response to standard therapy.^7,8^ *MYC* rearrangement (*MYC*-R) is a feature shared with BL, and such tumors often have some BL-like genomic features and patterns of gene expression.^9,10^ Gene expression profiling also has been used to distinguish DLBCL and BL,^11,12^ but intermediate categories of high-grade DLBCL remain, including those with double hits, those whose overall pattern of gene expression resembles that of BL, and those that strongly express both MYC and BCL2^13^ proteins, for which the optimal group definition and treatment choices are still unclear. These *MYC* and BL-related groups do not feature clearly in the recent genetic classifications,^5,6^ but they are present in two new WHO designations as high-grade B-cell lymphoma with *MYC* and *BCL2* and/or *BCL6* translocation and high-grade B-cell lymphoma not otherwise specified.^14^ The difficulty in defining the optimum approach to this group is partly explained by the low frequency of groups such as double-hit lymphomas, and the absence of a clear biologic definition. With the benefit of a large clinical trial data set, we suggest here a unifying definition of a molecular high-grade (MHG) class that is based on gene expression and propose that it should form part of our evolving understanding of DLBCL.

The Randomized Evaluation of Molecular-Guided Therapy for DLBCL With Bortezomib (REMoDL-B) clinical trial^15^ tested standard therapy for DLBCL (rituximab, cyclophosphamide, doxorubicin, vincristine, and prednisolone [R-CHOP]) against its combination with the proteasome inhibitor bortezomib (RB-CHOP). The hypothesis was that bortezomib indirectly inhibits the nuclear factor kappa-light-chain-enhancer of activated B cells pathway believed to be specifically active in the ABC variant.^16^ We present our analysis of the trial data focused on the MHG group by showing that a biologically coherent and distinctive group with significantly poorer prognosis can be identified and validated in independent data. We suggest that this group should be targeted in the future with precision medicine approaches.

## PATIENTS AND METHODS

### Data Set Summary

**FIG 1.**
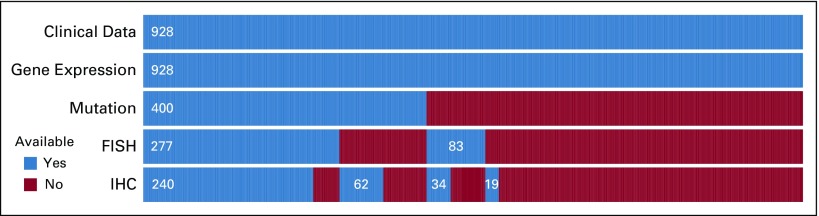
Data set summary. The analysis included 928 patients from the Randomized Evaluation of Molecular-Guided Therapy for Diffuse Large B-Cell Lymphoma With Bortezomib (REMoDL-B) clinical trial. All had full clinical data (diagnostic variables, treatment, treatment response, progression status, and follow-up time) and whole-genome expression profiling assayed by the DASL version 4 array (Illumina, San Diego, CA). A subset of 400 patients was analyzed for somatic mutations with a targeted 70-gene panel. In addition, 360 patients were tested for *MYC*, *BCL2*, and *BCL6* rearrangements by fluorescent in situ hybridization (FISH), and 355 patients were tested for MYC and BCL2 protein expression with immunohistochemistry (IHC).

A total of 928 patients treated in the REMoDL-B trial^15^ (Data Supplement) were included in this retrospective study. Genome-wide gene expression data were available for all patients from formalin-fixed paraffin-embedded tissue samples. A subset of 400 patient samples was sequenced for a 70-gene panel, chosen by known relevance to DLBCL, with HaloPlexHS (Agilent Technologies, Santa Clara, CA) target enrichment and HiSeq 4000 (Illumina, San Diego, CA) sequencing, and analyzed for somatic mutations (Data Supplement). Furthermore, for the purpose of comparison with other known biomarkers, a subset of 360 patient samples was tested for *MYC*, *BCL2*, and *BCL6* chromosomal rearrangements with fluorescent in situ hybridization assays, and a subset of 355 samples was tested for MYC and BCL2 protein expression with immunohistochemistry using tissue microarrays. Clinical features, treatment, progression status, and follow-up data (median, 30 months) were available for all patients. The available data are summarized in Figure 1, and full details are provided in the Data Supplement. Methodological detail in addition to that given here is provided in the Data Supplement.

### COO Classification and the MHG Subgroup

Gene expression data–based COO classification was performed in the trial with the DLBCL automatic classifier^17^ in real-time for random assignment to R-CHOP or RB-CHOP in the second to sixth treatment cycles. For this analysis, the COO classification was repeated with the same method to take advantage of higher-quality samples that became available for some patients after randomization and improved data normalization over the complete trial data set. The overall concordance between this retrospective COO classification (255 ABC, 543 GCB, and 130 unclassified [UNC]) and the real-time prospective classification (244 ABC, 475 GCB, and 199 UNC) from the trial randomization was 87%. The main change between prospective and retrospective COO DLBCL automatic classification was the reduction of UNC patients who were reassigned to ABC and GCB in the retrospective classification. The classification shift between GCB and ABC was low (4.5%). Full details of the prospective and retrospective classifications are provided in the Data Supplement.

Our previous work had shown that patients with DLBCL with a BL-like pattern of gene expression had poor prognosis.^18^ Accordingly, we applied the gene expression classifier developed in that work to REMoDL-B patients, using all those with BL and DLBCL in our local database for the normalization background, to define the MHG class. Of note, the definition of the MHG class was determined in previous work with an earlier data set and was not trained or determined in any way from the REMoDL-B trial data. Conventional diagnosis of all identified patients with MHG was checked for this study (Data Supplement) and indicated that this group had DLBCL by morphology and immunophenotype, thus excluding the possibility of contamination with patients with BL.

### External Validation

Although the MHG group was defined independently of the clinical trial data, we validated it further on another independent and recently published data set^4^ (European Genome-Phenome Archive study accession EGAS00001002606) by using the core set of 624 patients whose gene expression profiles were examined in that study by RNA sequencing. Adaptation of our classifier to these data was straightforward because the classifier was designed and tested^18^ for cross-platform applicability. We note, however, that there are no diagnoses of BL in this data set, which could have a minor effect on the overall normalization, and that in this data set four classifier genes (*BMP7*, *TCL6*, *SOX11*, and *C7orf10*) had very low estimated expression levels from the RNA sequencing data. The classifier, therefore, was retrained using the original training data,^18^ with the gene set reduced by these four genes for application to this data set. COO classification was that provided by the authors. Analysis of mutation frequencies in this data set used the 150 identified driver genes from the original article filtered by at least 5% mutation frequency in at least one subgroup and significantly different frequency (Fisher’s exact *P* < .05) between any two groups of MHG, GCB, and ABC.

### Statistical Analysis

All survival analyses were carried out using the survival package in R (https://cran.r-project.org), using single-factor and multivariable Cox proportional hazards regression models and likelihood ratio tests. Associations in count data related to clinical variables, chromosomal rearrangements, mutations, and so forth were analyzed with Fisher’s exact test. For continuous variables, differences between groups were tested with Mann-Whitney *U* or *t* tests as appropriate. All quoted *P* values are two-sided.

## RESULTS

### Definition and Clinical Outcome of MHG Lymphoma

**FIG 2.**
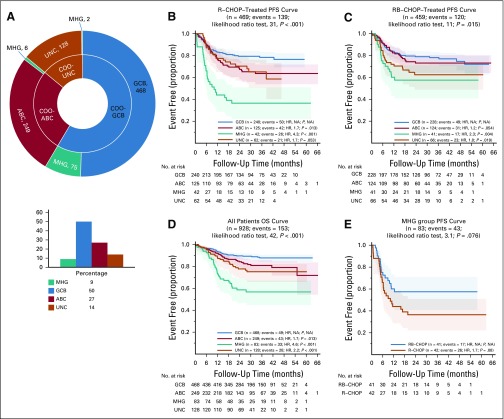
Retrospective Randomized Evaluation of Molecular-Guided Therapy for Diffuse Large B-Cell Lymphoma With Bortezomib (REMoDL-B) trial analysis with the molecular high-grade (MHG) group. (A) Number of REMoDL-B patients with standard cell-of-origin (COO) classification (inner circle) and with MHG patients separated into a new class (outer circle). For the latter, the overall distribution between classes is shown in the histogram. (B) Progression-free survival (PFS) curves for rituximab plus cyclophosphamide, doxorubicin, vincristine, and prednisone (R-CHOP)–treated patients by classification, with MHG as a separate class. (C) As in (B) but for patients treated with R-CHOP with the proteasome inhibitor bortezomib (RB-CHOP). (D) As in (B), but overall survival (OS) for all patients. (E) Progression-free survival for MHG patients separated by treatment. *P* values given for overall difference in survival are from the likelihood ratio test and for individual groups with reference to the best-surviving group from a Cox proportional hazards regression model. ABC, activated B-cell-like; GCB, germinal center B-cell-like; HR, hazard ratio; NA, not applicable; UNC, unclassified.

Our gene expression classifier assigned 83 REMoDL-B patients as MHG (9%; Fig 2). Seventy-five patients in the MHG group (90%) were within the original GCB group (Fig 2A) and were considered separately in the subsequent analysis, with GCB, ABC, and UNC referring (unless otherwise stated) to patients within those classes but not identified as MHG. A full analysis of associations between the COO and MHG groups and other clinical prognostic factors (Data Supplement) showed that MHG has a significantly higher International Prognostic Index^19^ (IPI; *P* = .004), tumor bulk (*P* = .007), and stage (*P* = .06). Median lactate dehydrogenase levels in patients with MHG were higher by almost 1.5-fold (*P* < .001), which reflects higher proliferation and cell turnover.

Significant differences in progression-free survival (PFS) and overall survival were observed between MHG and other COO groups. After treatment with R-CHOP, three-year PFS rate estimates were 37% for MHG, 78% for GCB, 64% for ABC, and 65% for UNC (Fig 2B-2E). Multivariable Cox proportional hazards regression models were used to assess the additional prognostic information provided by the MHG group (Table 1). The first model showed that MHG provided additional information to that from clinical variables as encapsulated in the standard IPI (*P* < .001), and the second showed that MHG provided additional information to relevant clinical variables from the IPI and other COO groups. In the RB-CHOP arm (Fig 2C), the results in the MHG group showed a nonsignificant trend toward improvement (3-year PFS rate, 58%), which provides possible evidence of a positive effect of bortezomib despite the small number of patients (*P* = .08 Fig 2E).

**TABLE 1.**
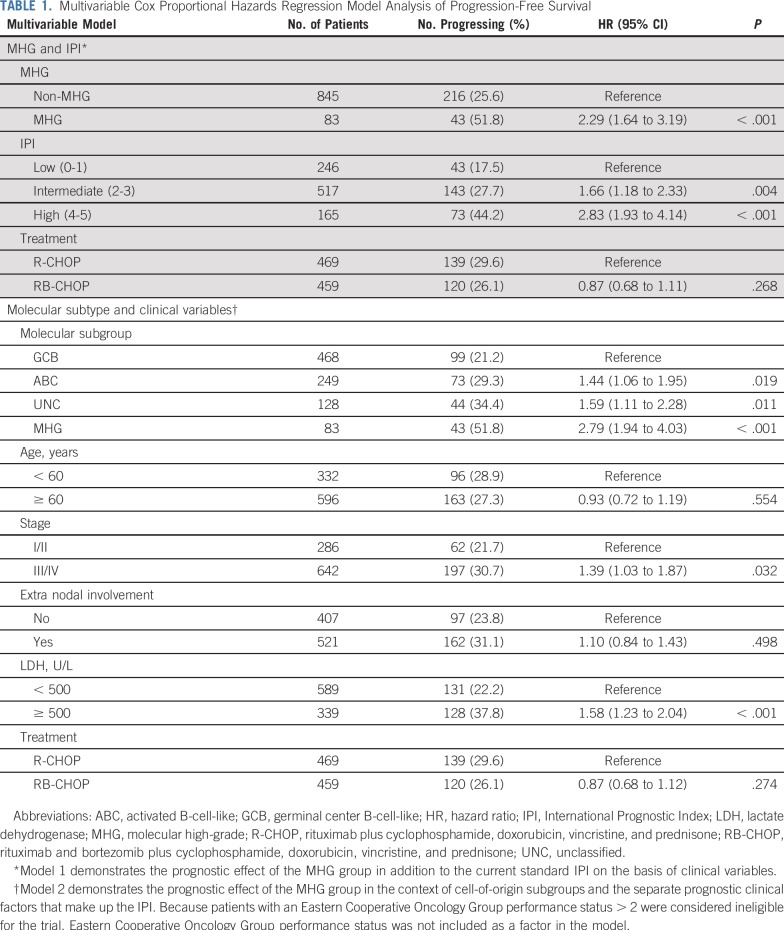
Multivariable Cox Proportional Hazards Regression Model Analysis of Progression-Free Survival

### Molecular Characteristics of the MHG Group

To clarify the molecular characteristics of the MHG group, we augmented the trial data set with 70 patients with confirmed BL from a previous study^18^ that used the same platform to measure gene expression (Data Supplement) and performed differential gene expression and gene expression signature analyses. Differential expression analysis (Data Supplement) revealed that BL is characterized by a large number of upregulated genes compared with both GCB (2,483 genes) and MHG (1,784 genes). In contrast, the comparison of MHG and GCB revealed only 382 upregulated genes. Downregulated genes had a similar pattern, and together, these figures indicate that MHG is an intermediate group but closer to GCB than to BL.

**FIG 3.**
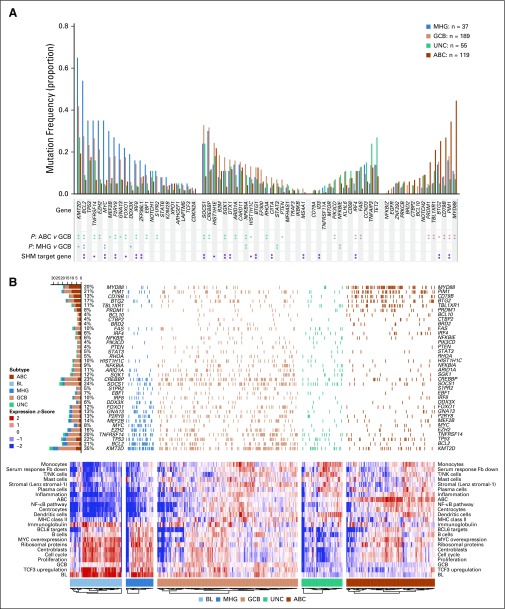
Molecular characteristics of the molecular high-grade (MHG) group. (A) Mutation frequencies for 400 Randomized Evaluation of Molecular-Guided Therapy for Diffuse Large B-Cell Lymphoma With Bortezomib (REMoDL-B) patients in MHG, germinal center B-cell-like (GCB), unclassified (UNC), and activated B-cell-like (ABC) subgroups for the 70-gene panel (statistical significance of differences at *P* < .05 [single asterisks]) and *P* < .01 [double asterisks] by Fisher’s exact test). Known (double asterisks) and predicted (single asterisks) aberrant somatic hypermutation (SHM) target genes from Schmitz et al.^6^ (B) Heat map of gene expression signatures (bottom) and associated mutations (top; limited to genes with mutation frequency > 5% in at least one group and significantly different [*P* < .05] between any two groups of MHG, GCB, and ABC). The heat map shows the mean gene expression level (red = high to blue = low) over genes in the chosen signature cluster representative and is augmented in 70 patients with Burkitt lymphoma (BL) for comparison of gene expression patterns. The left-side bar chart (top) recapitulates the incidence of the corresponding mutations and their distribution among subgroups. Fb, fibroblast; NA, not applicable; NF-κB, nuclear factor kappa-light-chain-enhancer of activated B cell; NK, natural killer cell.

Gene signature databases were used to obtain functional insights into MHG biology (Fig 3). Figure 3B shows the results from a compact and lymphoma-enriched database^20^ for patients with mutation data available (an analysis of all patients revealed the same patterns). To simplify the analysis, signatures were first clustered, each cluster was named according to the function of its constituent signatures and their genes, and expression values were plotted in the heat map for a chosen representative signature for each cluster. This shows that MHG and BL share high expression of signatures that contain cell cycle genes, ribosome biogenesis, *MYC* overexpression, and *TCF3* targets, which suggests a shared proliferative phenotype. Of note, BL and MHG showed high expression of the germinal center centroblast (dark zone) signature and lower expression of the germinal center centrocyte (light zone) signature relative to other subgroups.^21^ (We note that some patients with ABC showed relatively high expression of both centrocyte and centroblast signatures, which is likely due to cell cycle genes in the latter signature and may reflect proliferative ABCs that resemble plasmablasts.) Signatures that show low expression in MHG and BL include those involved with MHC class II, stromal, and immune response. Of note, our differential expression analysis shows that *FOXP1*, which has a number of functions, including the control of apoptotic genes, immune response signatures, and MHC class II,^22,23^ is upregulated in BL and MHG relative to GCB. A more comprehensive gene set enrichment analysis^24^ using MSigDB^25^ to analyze the differential expression gene lists (Data Supplement) confirmed these results.

Somatic mutation data (Fig 3A; Data Supplement) revealed the expected associations for the ABC and GCB groups,^26,27^ with the former enriched for mutations in *MYD88*, *PIM1*, *CD79B*, *BTG2*, *TBL1XR1*, and *PRDM1* and the latter for *BCL2*, *EZH2*, *KMT2D*, and *MEF2B*. MHG had significantly higher mutation frequencies than GCB in *KMT2D*, *BCL2*, *MYC*, and *DDX3X*, whereas some frequent mutations in GCB (eg, *B2M*, *SGK1*, *NFKBIA*) were rare in MHG. These mutation patterns share some features (*MYC*, *DDX3X*) but not all (*KMT2D*, *BCL2*) with BL.^28,29^ In a similar vein, MHG did not have a high rate of mutation of *TCF3* or its negative regulator *ID3* typical of BL.^28-30^ Expression of *ID3* was reduced in MHG compared with BL, whereas *TCF3* was expressed at similar levels (Data Supplement), which suggests that alternative regulatory mechanisms operate for these genes in MHG.

Aberrant somatic hypermutation^31^ probably explains the high mutation rates of *MYC* and *BCL2* in MHG. Of note, *MYC* mutations are associated with *MYC*-R within the MHG class (12 of 16 rearranged cases also are mutated) but not in other classes (only one of 12 rearranged cases is mutated), which suggests that *MYC-*Rs outside MHG are biologically different.

### Comparison With Other Established Biomarkers

**FIG 4.**
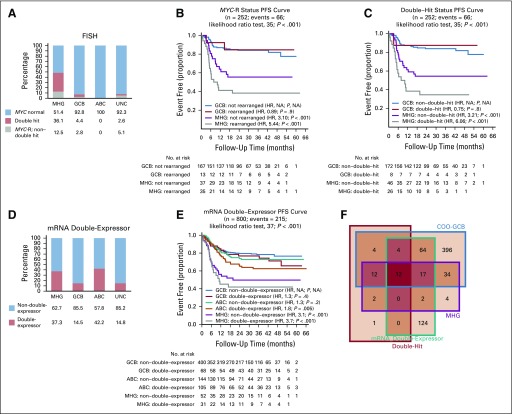
Molecular high-grade (MHG) group in relation to *MYC* rearrangement (*MYC*-R), double-hit, and double-expressor biomarkers. (A) The proportion of *MYC*-R, double-hit (*MYC*-R as well as *BCL2* and/or *BCL6* rearranged), and normal in each class as a percentage of those for which fluorescent in situ hybridization (FISH) data were produced. (B) Progression-free survival (PFS) by dividing MHG and germinal center B-cell-like (GCB) classes according to *MYC*-R status. (C) as in (B) by dividing according to double-hit status. (D) The percentage of mRNA double-expressor and normal status in each class. (E) PFS by dividing MHG, GCB, and activated B-cell-like (ABC) classes by double-expressor (mRNA) status. (F) Venn diagram showing overlaps among GCB, MHG, double-hit, and double-expressor groups (note that here, cell of origin [COO]-GCB is the original classification that includes patients with MHG and that some outside the double-hit category have not been tested for double-hit status). *P* values given for overall difference in PFS are from the likelihood ratio test and for individual groups with reference to best-surviving (GCB) group from a Cox proportional hazards regression model. HR, hazard ratio; NA, not applicable; UNC, unclassified.

We assessed the relationship of the MHG group to biomarkers commonly used to characterize related high-risk DLBCL (Fig 4). Of the 360 patients for whom fluorescent in situ hybridization data were available, 51 (14%) had *MYC*-R, and 35 of these (67%) were double-hit (also with *BCL2* and/or *BCL6* rearrangement). Most *MYC*-Rs (75%) were in the MHG group, with the remainder lying in GCB and UNC (MHG enrichment by Fisher’s exact test, *P* < .001), but only 48.6% and 36.1% of the MHG group were *MYC*-R and double-hit, respectively (Fig 4A).

Both patients with *MYC*-R and patients with double-hit status had worse PFS than those who were *MYC-*normal (Data Supplement). Furthermore, irrespective of *MYC*-R or double-hit status, the MHG group had a lower PFS than the GCB group (Figs 4B and 4C). Of note, although patient numbers were small in the GCB group, there is no evidence of an effect of *MYC*-R or double-hit status on PFS, but in MHG, both confer even lower PFS. A comparative gene expression analysis (Data Supplement) showed no differentially expressed genes between *MYC*-R and *MYC*-normal within the MHG group but did show 54 differentially expressed genes between *MYC*-R MHG and *MYC*-R GCB, which supports the biologic distinctiveness of MHG.

A subset of 355 samples also was investigated for MYC and BCL2 protein expression by IHC to identify cases of double-expressors, and at the mRNA level all trial samples were assessed for combined high expression of *MYC* and *BCL2*. In contrast to double-hits, double-expressors were found in significant numbers in all groups, including ABC (Figs 4D and 4F). Although protein expression was correlated with mRNA level (Data Supplement), the immunohistochemistry-identified double-expressor group did not show significant PFS separation in this study (Data Supplement). However, patients with double-expressor status defined by mRNA level where data covered all samples had a significantly lower PFS and further separated patients within subtypes (Fig 4E; Data Supplement). Patients with MHG lymphoma nevertheless showed worse outcome regardless of double-expressor status relative to ABC and GCB subgroups (Fig 4E). Details of the distribution of all biomarkers in the COO and MHG groups are provided in the Data Supplement as is a full analysis of the prognostic effects of these biomarkers.

### External Validation of the MHG Group

**FIG 5.**
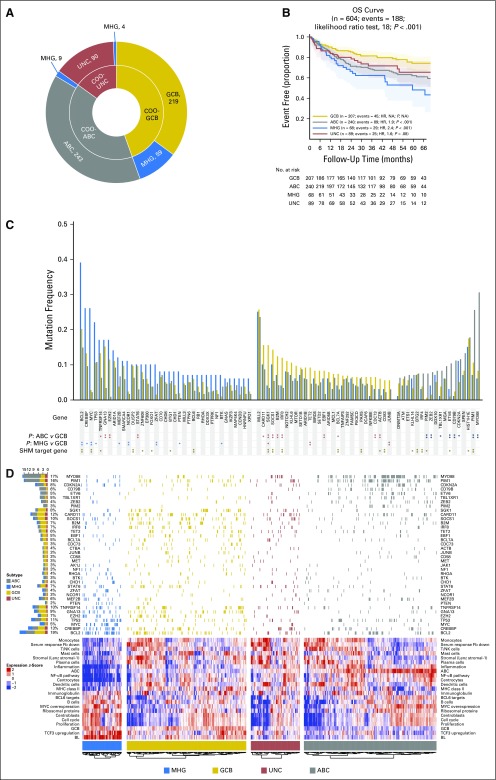
Validation of the molecular high-grade (MHG) group in an external data set. (A) Number of patients in each group, cell-of-origin (COO) classification (inner circle), and MHG classification of patients separated into a new group (outer circle). (B) Overall survival (OS) curves by group. (C) Mutation frequencies by group (statistical significance of differences at *P* < .05 [single asterisks] and *P* < 0.01 [double asterisks] by Fisher’s exact test). Known (double asterisks) and predicted (single asterisks) aberrant somatic hypermutation (SMH) target genes from Schmitz et al.^6^ (D) Gene expression signature heat map (bottom using the same signatures as Fig 3B) and mutations (top; limited to genes with mutation frequency > 5% in at least one group and significantly different [*P* < .05] between any two groups of MHG, germinal center B-cell-like [GCB], and activated B-cell-like [ABC]) for the patients analyzed. The heat map shows the mean gene expression level (red = high to blue = low) over genes in the chosen signature cluster representative. The left-sidebar chart (top) recapitulates the incidence of the corresponding mutations and their distribution among subgroups. BL, Burkitt lymphoma; Fb, fibroblast; HR, hazard ratio; NF-κB, nuclear factor kappa-light-chain-enhancer of activated B cell; NK, natural killer; UNC, unclassified.

Our analysis of the external validation data set^4^ is shown in Fig 5. Seventy-two of patients (11.5%) were classified as MHG, and consistent with the first data set (Fig 2A), the majority (82%) of the MHG group was derived from the GCB subtype (Fig 5A). The MHG group showed similar associations with clinical variables (Data Supplement) and similar mutation spectrum in the REMoDL-B and validation data sets (Figs 5C and 5D; Data Supplement). Gene expression signature analysis in the validation data set (Fig 5D), which used our signature set from Figure 3, or the authors’ signature set used in their original article (Data Supplement), showed the same proliferation and centroblast-related biology. MHG had a higher risk in these authors’ prognostic model compared with the remaining GCBs (*P* < .001) and a poor outcome, with a significantly lower overall survival than the other GCBs (*P* < .001; Fig 5B).

## DISCUSSION

We have defined the MHG group of patients with DLBCL that identifies a poor-risk subgroup primarily within the conventional GCB COO class. This encompasses most patients with double-hit lymphoma but extends the molecular identification to more than double the size of this poor-prognosis group, and significantly, this also reciprocally enriches the remaining patients with GCB DLBCL as a very good–prognosis group. Our analysis indicates that MHG is a robust and distinct group that is identifiable in independent data sets. MHG lymphoma has similarity in gene expression to both BL and GCB-DLBCL but with immunophenotype in keeping with DLBCL rather than BL and a characteristic pattern of genomic mutation. The poor prognosis for this group when treated with R-CHOP suggests that different approaches are required: either intensification of the type increasingly used for the double-hit lymphomas or, potentially, targeted agents that may preferentially affect more rapidly cycling cells. Gene expression patterns indicate that MHG has a highly proliferative phenotype and shares features with centroblasts of the germinal center dark zone in contrast to the centrocyte or light zone features of other GCBs.^21^

Recent analyses have suggested new taxonomies of DLBCL on the basis of genetic characteristics.^5,6^ Whereas Schmitz et al^6^ used a data set focused strongly on the ABC and UNC COO groups, where the MHG group would be under-represented, Chapuy et al^5^ commented on the genetic complexity of *MYC* and *BCL2* dysregulation that is represented in more than one of their clusters. Although there are limitations to our genetic data, which are based on a small gene panel without germline control and do not include copy number and other structural variations, our data reinforce the distinction among BL, MHG, GCB, and ABC. However, the reproducibly poor outcome in the MHG group suggests that in this case, the gene expression state captures biologically and clinically important features that are not readily identified from the use of genetics alone. Indeed, our mutation data suggest that the expression state may result from a number of different genetic drivers, including *MYC* and *BCL2* rearrangements, epigenetic effects related to mutations in *KMT2D*^32^ and *EZH2*, and mutations that affect other pathways. Although only a small number of *MYC*-Rs were identified outside the MHG group, their outcomes were similar to those of the other GCBs. Our data also indicate that *MYC* mutation levels of *MYC*-Rs differ between MHG and other groups, which suggests a different biology that could be related to different translocation partners.^33^

Evidence from the trial of a possible positive effect of bortezomib in the MHG group, although lacking statistical power, suggests a potential treatment option for this highly aggressive subtype. In future studies, it will be important to explore this mechanism, which seems unlikely to be mediated by the nuclear factor kappa-light-chain-enhancer of activated B cells pathway that is not believed to be active in GCB or MHG tumors.
